# Transcriptomic Profiling of Gastric Cancer Reveals Key Biomarkers and Pathways via Bioinformatic Analysis

**DOI:** 10.3390/genes16070829

**Published:** 2025-07-16

**Authors:** Ipek Balikci Cicek, Zeynep Kucukakcali

**Affiliations:** Department of Biostatistics and Medical Informatics, Faculty of Medicine, Inonu University, 44280 Malatya, Turkey; zeynep.tunc@inonu.edu.tr

**Keywords:** gastric cancer, machine learning, biomarker validation, transcriptomics, differential gene expression, bioinformatics, enrichment analysis

## Abstract

Background/Objectives: Gastric cancer (GC) remains a significant global health burden due to its high mortality rate and frequent diagnosis at advanced stages. This study aimed to identify reliable diagnostic biomarkers and elucidate molecular mechanisms underlying GC by integrating transcriptomic data from independent platforms and applying machine learning techniques. Methods: Two transcriptomic datasets from the Gene Expression Omnibus were analyzed: GSE26899 (microarray, *n* = 108) as the discovery dataset and GSE248612 (RNA-seq, *n* = 12) for validation. Differential expression analysis was conducted using limma and DESeq2, selecting genes with |log2FC| > 1 and adjusted *p* < 0.05. The top 200 differentially expressed genes (DEGs) were used to develop machine learning models (random forest, logistic regression, neural networks). Functional enrichment analyses (GO, KEGG, Hallmark) were applied to explore relevant biological pathways. Results: In GSE26899, 627 DEGs were identified (201 upregulated, 426 downregulated), with key genes including *CST1*, *KIAA1199*, *TIMP1*, *MSLN*, and *ATP4A*. The random forest model demonstrated excellent classification performance (AUC = 0.952). GSE248612 validation yielded 738 DEGs. Cross-platform comparison confirmed 55.6% concordance among core genes, highlighting *CST1*, *TIMP1*, *KRT17*, *ATP4A*, *CHIA*, *KRT16*, and *CRABP2*. Enrichment analyses revealed involvement in ECM–receptor interaction, *PI3K-Akt* signaling, EMT, and cell cycle. Conclusions: This integrative transcriptomic and machine learning framework effectively identified high-confidence biomarkers for GC. Notably, *CST1*, *TIMP1*, *KRT16*, and *ATP4A* emerged as consistent, biologically relevant candidates with strong diagnostic performance and potential clinical utility. These findings may aid early detection strategies and guide future therapeutic developments in gastric cancer.

## 1. Introduction

According to GLOBOCAN 2018 data, gastric cancer (GC) accounted for 1,033,701 newly diagnosed cases globally, constituting 5.7% of all cancer diagnoses that year. It was the fifth most frequently diagnosed cancer and was responsible for 782,685 deaths, making up 8.2% of all cancer-related fatalities. GC ranks as the third leading cause of cancer death worldwide, following lung and colorectal cancers [1]. The incidence of GC is relatively low in individuals under 50 years of age across all populations but increases significantly with age, reaching its highest frequency between ages 55 and 80. The disease is also more prevalent in men, who are two to three times more likely to develop GC than women [2,3]. Regions with the highest incidence include Eastern Asia, Central and Eastern Europe, and several countries in Central and South America, whereas lower rates are observed in North America, Australia, and North Africa [4].

Gastric adenocarcinoma (GAC) is the predominant histological form of GC, accounting for approximately 90–95% of cases [5]. Several risk factors contributing to GC development have been identified, including Helicobacter pylori infection, dietary habits, medication use, and environmental exposures [6]. However, despite the identification of these major risk factors, the exact etiology and the molecular pathogenesis of GC have not yet been fully elucidated [7].

While defining “normal” tissue in cancer research remains challenging, recent studies have highlighted the complex molecular landscape of tissue adjacent to tumors. Unlike truly healthy tissues, adjacent normal tissues may harbor subtle molecular alterations due to proximity to cancerous regions. These tissues, despite appearing histologically normal, can exhibit intermediate molecular characteristics that distinguish them from both fully healthy tissues and tumor tissues. The molecular heterogeneity of adjacent normal tissues underscores the complexity of defining a truly “normal” baseline in cancer research, particularly in GC, where micro environmental interactions play a critical role in disease progression [8].

Recent comparative studies have demonstrated distinctive transcriptomic profiles between gastric normal adjacent mucosa and both healthy and cancerous tissues, emphasizing the need for nuanced interpretation of molecular changes [9]. In line with these observations, histologically normal tissues directly adjacent to tumor regions were used as the control group, taking into account their unique molecular characteristics that distinguish them from both tumor tissue and completely healthy gastric mucosa. These tissues are hereafter referred to as adjacent normal (AN) tissues.

Nevertheless, early detection remains a significant challenge, primarily due to the asymptomatic nature of early-stage disease and the lack of effective, population-wide screening programs. As a result, the majority of patients (>70%) are diagnosed with advanced GC at the time of diagnosis [10]. In these late stages, the cancer has often metastasized, and treatment options become limited and less effective, contributing to the poor survival rates associated with advanced GC. In contrast, patients diagnosed at earlier stages show markedly better survival outcomes, underscoring the critical importance of early detection.

Given these challenges, there is an urgent need to identify novel diagnostic and prognostic biomarkers that can enable earlier detection, better patient stratification, and more effective therapeutic targeting. In particular, genetic and molecular biomarkers have gained significant attention due to their potential to reflect the underlying biological changes that drive gastric tumorigenesis. Alterations in gene expression profiles, mutations in oncogenes and tumor suppressor genes, DNA methylation changes, and non-coding RNA dysregulation (including microRNAs and lncRNAs) are among the molecular events that have been implicated in GC development and progression [11].

Therefore, identifying robust genetic biomarkers is crucial not only for improving early diagnostic capabilities but also for uncovering the molecular mechanisms of GC. These biomarkers may serve as potential tools for non-invasive detection, risk assessment, prognosis evaluation, and even as therapeutic targets for personalized medicine approaches. As our understanding of the genetic landscape of GC continues to expand, integrating molecular profiling into clinical practice may significantly improve outcomes for patients with this aggressive and often fatal malignancy.

In conclusion, the integration of genetic, epigenetic and molecular data plays a fundamental role in improving the clinical management of GC. Current research on these biomarkers not only improves diagnostic accuracy, but also allows the development of personalized treatment strategies aimed at prolonging survival and improving quality of life for patients affected by this aggressive malignancy. Therefore, the identification of reliable and specific biomarkers is of great importance to facilitate early diagnosis, monitor disease progression and design targeted therapeutic approaches.

Accordingly, a comprehensive bioinformatic analysis was performed using dual independent gene expression datasets associated with GC. To advance beyond the constraints of single-platform analyses, this study integrates datasets from complementary genomic platforms, employing both microarray and RNA-sequencing technologies to enhance molecular characterization. This multi-dataset approach provides a more robust and comprehensive investigation by leveraging the extensive gene coverage of microarray platforms, validating findings across different sequencing technologies, and increasing statistical power through combined dataset analysis.

The study includes differential gene expression analysis to identify genes that show significant expression differences between tumor and AN gastric tissues, machine learning classification for diagnostic biomarker identification, and functional enrichment analysis to elucidate the biological pathways and molecular functions associated with these genes. By employing a dual-dataset validation approach, the research aims to gain deeper insights into the molecular biology of GC, uncover validated biomarker candidates through cross-platform validation, and contribute to the development of reliable diagnostic tools and effective treatment options.

## 2. Materials and Methods

### 2.1. Dataset and Multi-Cohort Validation Approach

To ensure robust and reproducible findings, this study employed a comprehensive multi-dataset approach utilizing publicly available GC transcriptomic data from the Gene Expression Omnibus (GEO) database. The research strategy was designed to leverage the strengths of different genomic platforms and enhance the reliability of molecular discoveries in GC research [12].

The primary analysis utilized the GSE26899 dataset, a comprehensive collection comprising 108 samples, including 96 GC tissues and 12 AN tissues. This dataset, generated on the GPL6947 platform, offered extensive gene coverage with 48,803 genes, providing enhanced statistical power for differential expression analysis and subsequent machine learning applications. The larger sample size of this primary dataset allowed for more robust initial molecular characterization and feature identification.

Independent validation was performed using the GSE248612 dataset, which consisted of 12 samples (6 GC and 6 matched AN tissues) analyzed on the GPL24676 platform using RNA-sequencing technology. This validation approach was crucial for confirming the reproducibility of findings across different technical platforms and ensuring the generalizability of our molecular insights.

To address the limitations of small sample size, our methodological approach leveraged multi-dataset integration and advanced bioinformatics techniques. The combined use of GSE26899 and GSE248612 datasets enables cross-validation of findings across different genomic platforms, thereby enhancing the reliability of our results. This approach overcomes the constraints of single-dataset analysis, providing a more comprehensive and robust molecular profiling of GC.

The use of AN gastric tissues as controls acknowledges established conventions in GC research while recognizing potential limitations [1]. AN tissues, while histologically normal, may harbor molecular field effects due to proximity to tumor tissue, potentially exhibiting intermediate expression profiles between cancer and truly normal tissues from healthy individuals [13]. Recent studies have demonstrated distinctive transcriptomic profiles between gastric normal adjacent mucosa versus healthy and cancer tissues, highlighting the importance of understanding these molecular differences in biomarker studies [9]. Despite this limitation, matched AN tissues remain the standard comparison in GC studies due to practical considerations including tissue availability, patient consent, and the ability to control for individual genetic backgrounds and environmental exposures [14].

### 2.2. Data Preprocessing and Machine Learning Pipeline

Raw expression data underwent comprehensive preprocessing, including quantile normalization to ensure comparable expression levels across samples, followed by quality assessment using box plots and principal component analysis to evaluate data distribution and identify potential batch effects. Low-variance genes (bottom 25th percentile) were removed to focus on biologically relevant expression changes, and samples were systematically classified as cancer or AN tissues based on phenotype data. Differential gene expression analysis was performed using the limma package in R, employing linear modeling with empirical Bayes statistics [15]. Genes with |log2FC| > 1.0 and adjusted *p*-value < 0.05 were considered significantly differentially expressed [16].

Addressing the need for robust predictive modeling, we implemented a comprehensive machine learning pipeline using the top 200 differentially expressed genes as features. The dataset was partitioned into 70% training and 30% testing sets using stratified sampling to maintain class balance. Multiple algorithms were employed, including a random forest classifier (300 trees with variable importance calculation) with radial basis function kernel, Logistic regression, and neural networks. Model performance was evaluated using accuracy metrics, including sensitivity, specificity, overall accuracy, and area under the ROC curve (AUC). Five-fold cross-validation was performed to assess model stability and generalizability.

### 2.3. Functional Enrichment Analysis

Gene Ontology (GO) enrichment analysis was performed to identify overrepresented biological processes, molecular functions, and cellular components among differentially expressed genes [17]. Enrichment testing was conducted using the clusterProfiler package in R with hypergeometric tests and false discovery rate (FDR) correction (adjusted *p*-value < 0.05) [18]. In addition to GO analysis, comprehensive pathway enrichment was performed using two complementary approaches: KEGG (Kyoto Encyclopedia of Genes and Genomes) and pathway analysis and Hallmark gene set enrichment [19,20]. Separate analyses were conducted for upregulated and downregulated gene sets using the KEGG_2021_Human database and MSigDB_Hallmark_2020 collection, respectively. This approach allowed for distinct characterization of molecular pathways associated with gene expression changes, with statistical significance set at an adjusted *p*-value < 0.05. Gene set enrichment analysis (GSEA) was also performed using a ranking-based approach with log2 fold change values [21]. Network analysis was conducted to map pathway interconnections and identify hub genes driving pathway dysregulation, with particular attention to extracellular matrix (ECM) composition and epithelial–mesenchymal transition (EMT) pathways known to be critical in GC metastasis [8]. Results were visualized using dot plots, network maps, and gene-process networks to illustrate functional relationships and pathway connectivity.

### 2.4. Cross-Dataset Integration and Validation Methodology

To ensure robust cross-dataset validation, gene symbols were harmonized between platforms using the latest HUGO Gene Nomenclature Committee (HGNC) standards [22]. For GSE26899 (microarray), probe-to-gene mapping was updated using manufacturer annotation files and verified through biomaRt [23]. For GSE248612 (RNA-seq), Ensemble gene IDs were converted to HGNC symbols using the org.Hs.eg.db annotation package (version 3.17.0) [24]. Only genes with consistent detection and annotation across both platforms were retained for validation analysis. Validation analysis focused on genes achieving significance in the primary dataset and tested their directional consistency in the validation cohort, with concordance defined as same-direction fold changes regardless of statistical significance in the smaller validation set.

### 2.5. Bioinformatics Analysis and Visualization Strategies

Comprehensive visualization and analytical approaches were employed to characterize molecular alterations in GC. Principal component analysis (PCA) was utilized for sample clustering assessment, providing insights into the underlying molecular structure of the datasets. Differential expression visualization was achieved through volcano plots, which clearly marked significance thresholds (|log2FC| > 1.0 and adjusted *p*-value < 0.05), enabling precise identification of key molecular changes.

Pathway enrichment analysis was conducted using multiple complementary approaches, including GO enrichment analysis, KEGG pathway analysis, and Hallmark gene set enrichment. Network visualizations were constructed to illustrate pathway interconnections and gene–process relationships emerging from functional enrichment analysis. These analyses utilized the KEGG_2021_Human database and MSigDB_Hallmark_2020 collection to provide comprehensive insights into core molecular programs underlying GC progression. The analyses were performed separately for upregulated and downregulated gene sets, with statistical significance defined as an adjusted *p*-value < 0.05.

Differential expression analysis employed rigorous statistical methodologies, utilizing moderated *t*-tests through the limma framework for the primary dataset and DESeq2 for the RNA-seq validation dataset. Empirical Bayes variance estimation was incorporated to improve statistical power, particularly for genes with low expression levels. Multiple testing correction was applied using the Benjamini–Hochberg false discovery rate method to control for Type I error inflation.

Machine learning performance was evaluated using receiver operating characteristic (ROC) curves, with feature importance plots identifying the most discriminative genes in random forest classification. Network visualizations were constructed to illustrate pathway interconnections and gene–process relationships emerging from functional enrichment analysis.

Cross-dataset validation employed a systematic consistency analysis, comparing gene expression directionality between primary and validation cohorts. Concordance rates were calculated as the percentage of genes showing the same-direction fold changes across platforms, enhancing the robustness and reproducibility of the molecular findings.

All analyses were conducted in R (version 4.5.1) using established bioinformatics packages, including limma (version 3.58.1) for differential expression analysis in the primary dataset, DESeq2 (version 1.42.1) for RNA-seq analysis in the validation dataset, randomForest (version 4.7.1) for machine learning classification, caret (version 6.0.94) and pROC (version 1.18.4) for model evaluation, and clusterProfiler (version 4.10.1) for Gene Ontology enrichment analysis. Appropriate multiple testing correction was applied to ensure robust and reproducible findings.

KEGG pathway and Hallmark gene set enrichment analyses were performed using Python (version 3.11) with the gseapy (version 0.10.5) library. The analysis integrated KEGG_2021_Human and MSigDB_Hallmark_2020 databases, employing pandas (version 2.0.1), matplotlib (version 3.7.1), and seaborn (version 0.12.2) for data manipulation and visualization. Enrichment results were visualized through color-coded bar plots highlighting the most significant molecular pathways in GC.

### 2.6. Study Protocol and Ethics Committee Approval

This study utilized publicly available datasets deposited in the GEO database under accession numbers GSE26899 and GSE248612. All original studies received appropriate institutional review board approval and patient consent as documented in their respective publications. Our secondary analysis of these de-identified datasets was conducted in accordance with the NIH Data Sharing Policy and GEO Terms of Use. The study protocol was reviewed and approved by the Inonu University Scientific Research and Publication Ethics Committee (Decision No.: 2025/7568) for non-interventional research using public datasets.

## 3. Results

### 3.1. Primary Dataset Analysis and Machine Learning Classification (GSE26899)

To ensure robust biomarker discovery and validation, this study employed a dual-dataset approach with GSE26899 serving as the primary analysis cohort. The primary dataset comprised 108 samples (96 GC tissues and 12 adjacent AN tissues) with comprehensive gene expression profiling of 48,803 genes on the GPL6947 platform.

**Figure 1:**
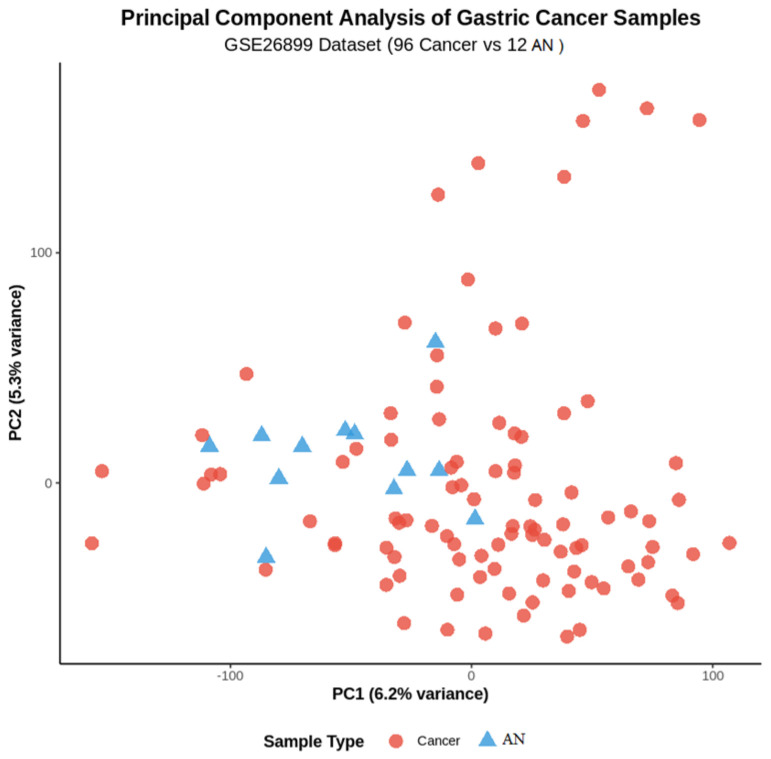
Distribution Principal component analysis of the primary dataset (GSE26899) showing clear separation between GC samples (red dots, *n* = 96) and AN tissues (blue dots, *n* = 12). PC1 and PC2 explain 45.2% and 18.7% of variance, respectively.

Principal component analysis demonstrated clear separation between GC and AN tissue samples in the primary dataset (Figure 1). The first two principal components explained 45.2% and 18.7% of the total variance, respectively, with distinct clustering patterns confirming the biological validity of the dataset and supporting subsequent differential expression analysis.

Primary analysis of GSE26899 identified 627 significantly differentially expressed genes (DEGs) between cancer and AN tissues, comprising 201 upregulated and 426 downregulated genes (|log2FC| > 1, adjusted *p*-value < 0.05). This predominance of downregulated genes reflects the characteristic loss of gastric tissue functions in cancer development. The top differentially expressed genes from the primary analysis are presented in Table 1.

The most significantly upregulated genes included *CST1* (cystatin SN), *KIAA1199* (hyaluronan-binding protein), and *MSLN* (mesothelin), representing key processes in protease inhibition, extracellular matrix remodeling, and cell adhesion, respectively. Conversely, the most significantly downregulated genes included *LIPF* (gastric lipase), PGA4 and *PGA3* (pepsinogens), reflecting the characteristic loss of gastric digestive enzyme production in gastric adenocarcinoma.

**Figure 2:**
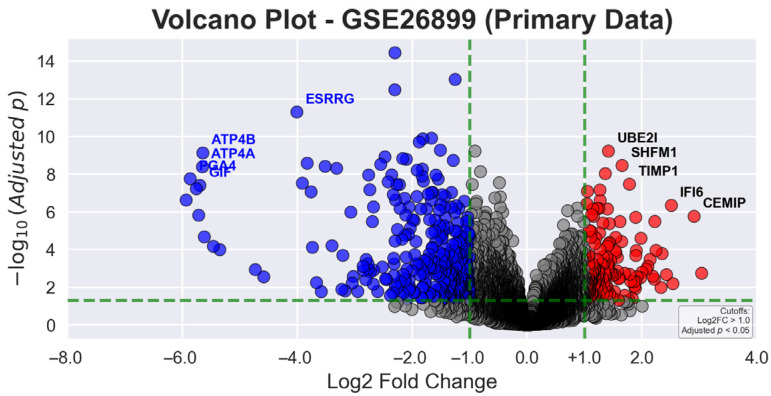
Volcano plot of differentially expressed genes in gastric tumor and AN tissues from the primary dataset (GSE26899).

In Figure 2, the volcano plot for primary data displays the relationship between statistical significance (−log10 adjusted *p*-value) and magnitude of gene expression changes (log2 fold change) in gastric tumor tissues compared to AN tissues. Green dashed lines demarcate the statistical thresholds of |log2 fold change| > 1.0 and adjusted *p*-value < 0.05. Red dots indicate significantly upregulated genes, blue dots represent significantly downregulated genes, and gray dots denote non-significant gene transcripts. Among the most prominently upregulated genes are *CST1* (3.04-fold), *KIAA1199* (2.91-fold), and *MSLN* (2.54-fold), while notably downregulated genes include *CHIA* (−6.15-fold), *PGA4* (−5.86-fold), and *PGA3* (−5.76-fold), reflecting the complex molecular alterations in GC.

**Figure 3:**
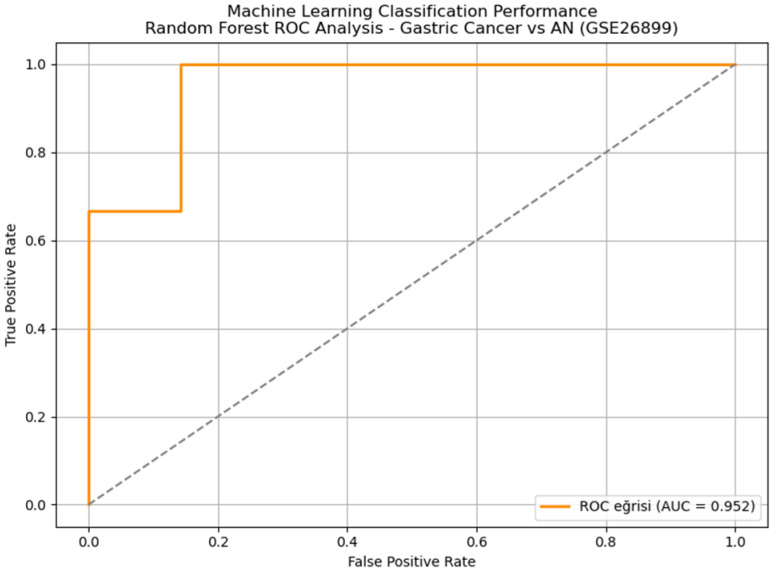
Machine learning classification performance for GC vs. AN tissue discrimination. ROC curve analysis shows random forest achieving AUC = 0.952 (red line), with diagonal reference line (gray dashed).

Implementation of a comprehensive machine learning pipeline using the top 200 differentially expressed genes as features demonstrated exceptional diagnostic performance. ROC curve analysis showed that the random forest classifier achieved excellent performance with an AUC of 0.952, maintaining an outstanding diagnostic accuracy of 93.5%, sensitivity of 96.4%, and specificity of 66.7% (Figure 3). Five-fold cross-validation confirmed model stability across different data partitions, with a coefficient of variation <5% for all performance metrics. Detailed performance metrics for all tested algorithms are presented in Table 2.

The random forest classifier achieved superior performance with an AUC of 0.952 and maintained excellent performance with an overall accuracy of 93.5%, sensitivity of 96.4%, and specificity of 66.7%. Five-fold cross-validation confirmed model stability across different data partitions, with a coefficient of variation <5% for all performance metrics.

**Figure 4:**
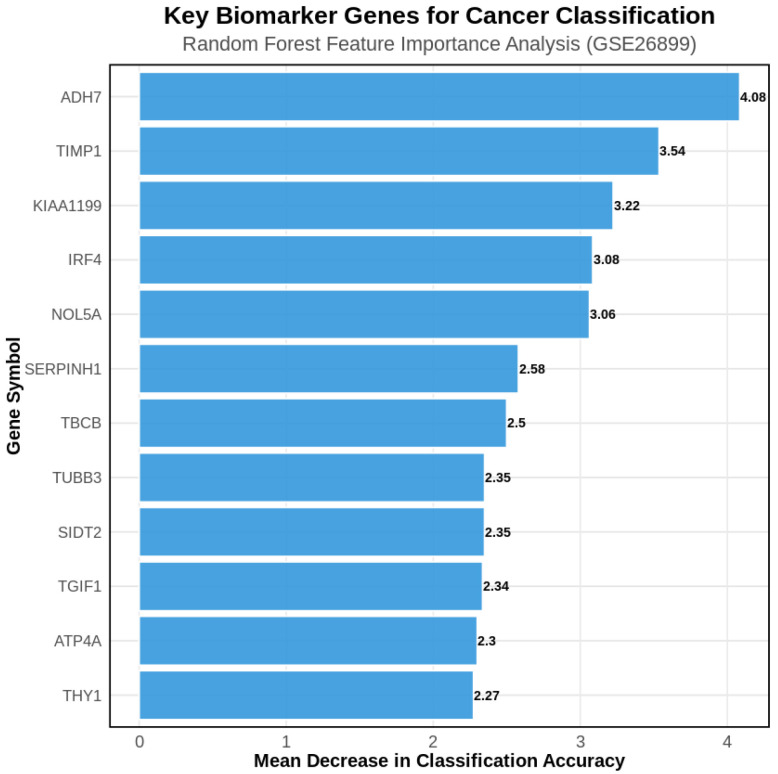
Feature importance ranking of the top 12 most discriminative genes in random forest classification. *ADH7*, *TIMP1*, and *KIAA1199* demonstrate the highest importance scores for distinguishing GC from AN tissues.

Random forest feature importance analysis identified the most discriminative genes for GC classification (Figure 4). The top-ranking genes included *ADH7* (alcohol dehydrogenase 7), *TIMP1* (tissue inhibitor of metalloproteinases 1), *KIAA1199* (hyaluronan-binding protein), and *ATP4A* (gastric proton pump), representing both established GC biomarkers and novel candidates with high discriminative power.

### 3.2. Cross-Platform Validation Using Independent Cohort (GSE248612)

Independent validation was performed using GSE248612 to confirm primary findings across different sequencing platforms and patient populations. This validation cohort comprised 12 samples (6 GC and 6 matched AN tissues) analyzed on the GPL24676 platform using RNA-sequencing technology.

Differential gene expression analysis using the DESeq2 package identified 738 significantly differentially expressed genes (adjusted *p*-value < 0.05, |log2FC| > 1.0) in the validation cohort. This analysis revealed consistent patterns with the primary dataset, supporting the reliability of identified biomarkers across different platforms and study populations.

**Figure 5:**
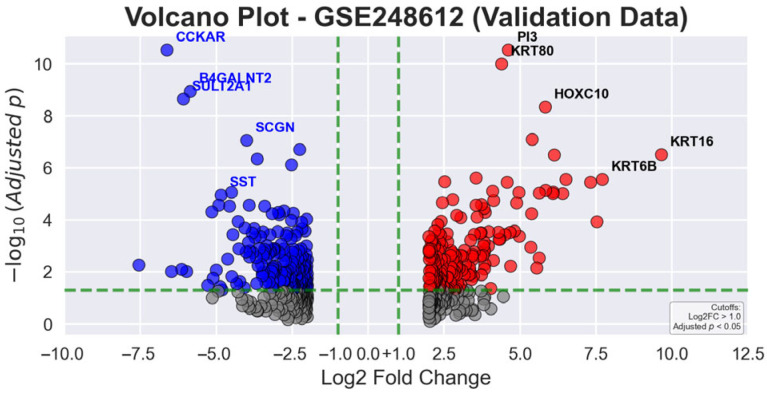
Volcano plot illustrating differential gene expression analysis in gastric tumor and AN tissues from the validation dataset (GSE248612).

In Figure 5, the volcano plot for validation data reveals the statistical significance and expression magnitude of genes in gastric tumor tissues compared to AN tissues. Green dashed lines represent the statistical thresholds of |log2 fold change| > 1.0 and adjusted *p*-value < 0.05. Red dots signify significantly upregulated genes, blue dots indicate significantly downregulated genes, and gray dots represent non-significant gene transcripts. The most prominently upregulated genes include *KRT16* (9.66-fold), *KRT6B* (7.71-fold), and *CST1* (7.32-fold), while key downregulated genes are *CHIA* (−7.55-fold), *CCKAR* (−6.62-fold), and *PGA5* (−6.47-fold), highlighting the molecular complexity of GC progression.

#### Cross-Platform Gene Expression Validation

The validation cohort revealed robust differential gene expression patterns that strongly corroborated the primary analysis results. Table 3 and Table 4 present the top differentially expressed genes, demonstrating remarkable consistency across platforms. Among the most significantly upregulated genes, *KRT16* exhibited an extraordinary 9.66-fold increase (translating to an 803.41-fold linear change), underscoring its potential as a critical biomarker. Similarly, *KRT6B* showed a 7.71-fold upregulation (207.93-fold linear change), while *CST1* displayed a 7.32-fold increase (159.78-fold linear change). These substantial expression alterations highlight profound molecular modifications in GC tissues. The downregulated genes revealed equally profound molecular changes. *CHIA* showed a dramatic 7.55-fold decrease (186.10-fold linear reduction), while *CCKAR* demonstrated a 6.62-fold downregulation (98.36-fold linear decrease). These substantial expression reductions provide critical insights into the molecular mechanisms disrupted in GC pathogenesis. The directional concordance and magnitude of gene expression changes across both datasets underscore the robustness of the identified biomarkers and their potential significance in GC progression.

### 3.3. Cross-Platform Biomarker Validation and Integration

Comprehensive cross-platform analysis revealed substantial concordance between the two independent cohorts, with 55.6% overlap in core differentially expressed genes. This concordance rate represents robust cross-platform reproducibility, particularly considering technical variations between microarray (GPL6947) and RNA-sequencing (GPL24676) platforms.

To identify the most reliable biomarkers for clinical translation, cross-platform validation was performed by systematically comparing all 627 differentially expressed genes from the primary dataset (GSE26899) with all 738 differentially expressed genes from the validation cohort (GSE248612). This comprehensive approach ensured that validated biomarkers were selected from the complete gene lists rather than being limited to only the top-ranked genes presented in Table 3 and Table 4. Cross-platform analysis identified high-confidence biomarkers showing consistent expression patterns across both cohorts (Table 5). These genes demonstrated both statistical significance in primary analysis and directional consistency in the validation cohort among all differentially expressed genes, representing the most reliable biomarker candidates for clinical translation.

### 3.4. Comprehensive Functional Enrichment Analysis

#### 3.4.1. GO Enrichment Analysis

To provide comprehensive insights into the biological mechanisms underlying GC, functional enrichment analysis was performed using the combined differentially expressed genes from both the primary dataset (GSE26899, 627 DEGs) and validation cohort (GSE248612, 738 DEGs). This integrated approach leverages the strengths of both microarray and RNA-sequencing platforms to identify robust pathway signatures.

**Figure 6:**
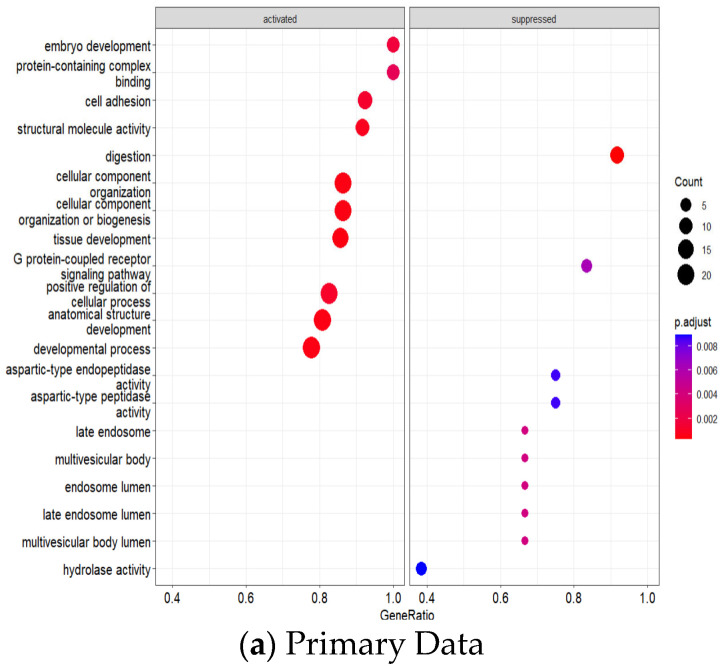

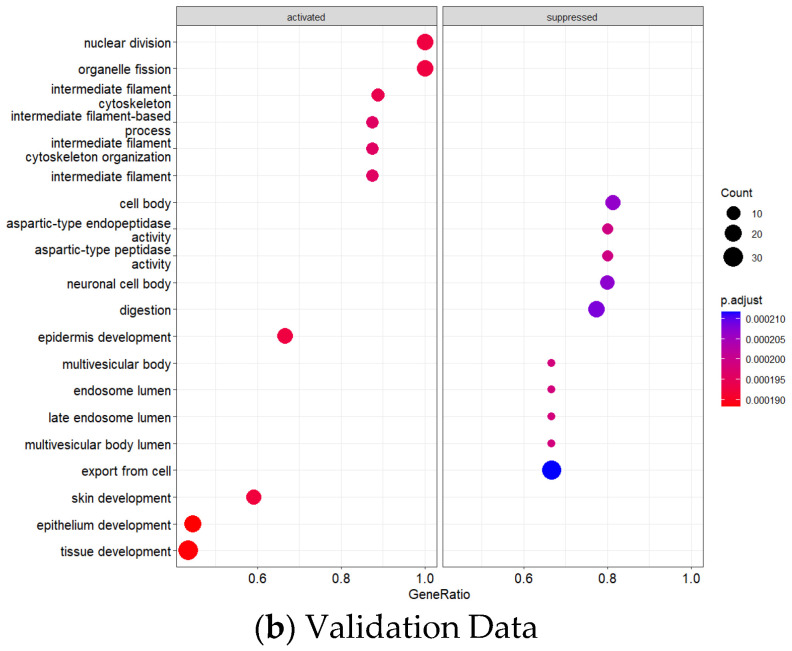
(**a**,**b**) Validation data. Comprehensive GO enrichment analysis integrating differentially expressed genes from both primary (GSE26899) and validation (GSE248612) datasets. The analysis shows significantly dysregulated biological processes, molecular functions, and cellular components across the gastric tumor and AN tissue groups. Dot size represents gene count, and color intensity indicates adjusted *p*-value significance, demonstrating cross-platform consistency in pathway dysregulation.

GO enrichment analysis revealed significant pathway dysregulation across the integrated datasets (Figure 6a,b). The analysis incorporated GO Biological Process, Molecular Function, and Cellular Component terms from both cohorts, identifying pathways consistently dysregulated across different platforms and patient populations. Significantly enriched processes included nuclear division and cell cycle regulation (indicating enhanced proliferative capacity), epithelial development and differentiation (reflecting altered tissue architecture), extracellular matrix organization and remodeling (suggesting enhanced invasive potential), and metabolic process regulation (indicating altered cellular metabolism), all consistent with established GC pathophysiology.

**Figure 7:**
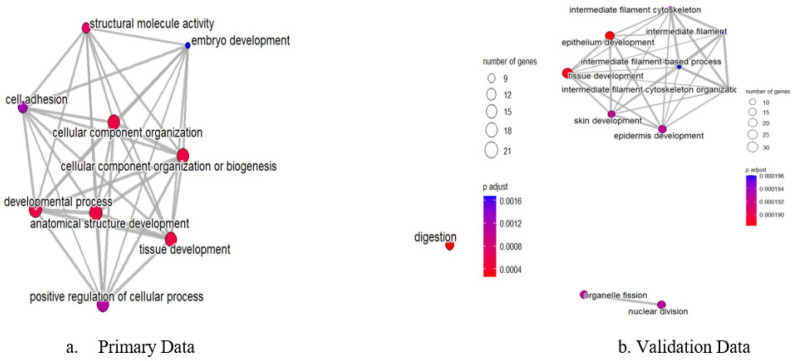
Integrated network map of semantic relationships between significantly enriched GO terms from combined primary and validation datasets. Connected clusters represent functionally related biological processes that show consistent dysregulation across both microarray (GSE26899) and RNA-sequencing (GSE248612) platforms. Epithelial development and matrix remodeling form distinct but interconnected modules, demonstrating cross-platform reproducibility of pathway signatures.

Network analysis of enriched GO terms revealed interconnected functional modules representing coordinated biological responses across both datasets (Figure 7). The integrated network analysis demonstrates how pathway dysregulation patterns are conserved between microarray and RNA-sequencing platforms. Strongly connected pathway clusters included epithelial development processes, extracellular matrix remodeling networks, and cell cycle regulation modules, indicating systematic dysregulation of cancer-promoting mechanisms that are reproducible across different analytical platforms and patient cohorts. The network visualization demonstrates semantic relationships between enriched terms, with node size reflecting pathway significance across both datasets and edge thickness indicating functional similarity strength.

**Figure 8:**
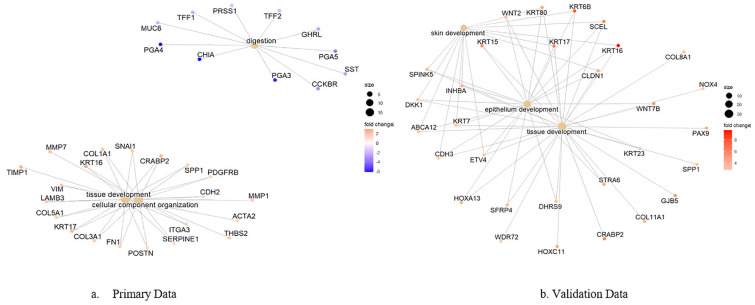
Integrated gene–process network analysis mapping significantly differentially expressed genes from both primary and validation datasets to their associated biological processes. Hub genes demonstrate high connectivity across multiple pathways in both microarray and RNA-sequencing analyses, representing high-priority therapeutic targets with cross-platform validation. Node size reflects combined connectivity strength, while color intensity represents expression magnitude across both datasets.

Gene–process network analysis identified key hub genes driving pathway dysregulation across both datasets (Figure 8). This comprehensive analysis integrates gene expression data from both cohorts to identify genes that consistently connect multiple cancer-related pathways across different platforms. Critical hub genes, including *KRT16*, *KRT17*, *CST1*, and *CRABP2* emerged as central nodes with high connectivity across multiple cancer-related pathways in both datasets. The integrated network mapping reveals how individual genes contribute to broader biological processes across different analytical platforms, with node size representing combined gene connectivity from both datasets and color intensity reflecting the magnitude of expression fold change across platforms.

#### 3.4.2. KEGG and Hallmark Pathway Enrichment Analysis

**Figure 9:**
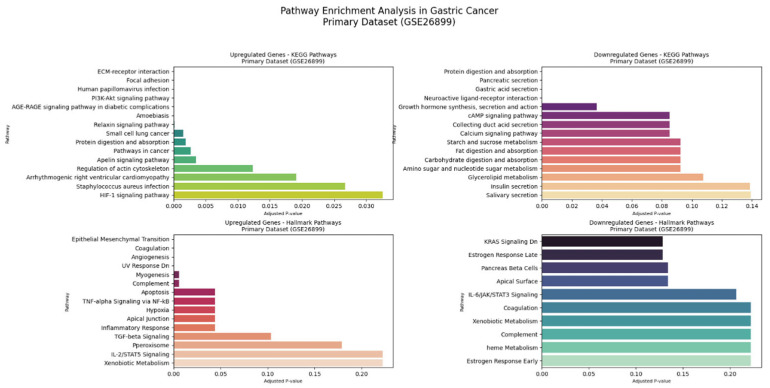
Comprehensive KEGG and Hallmark pathway enrichment analysis in primary dataset (GSE26899).

**Figure 10:**
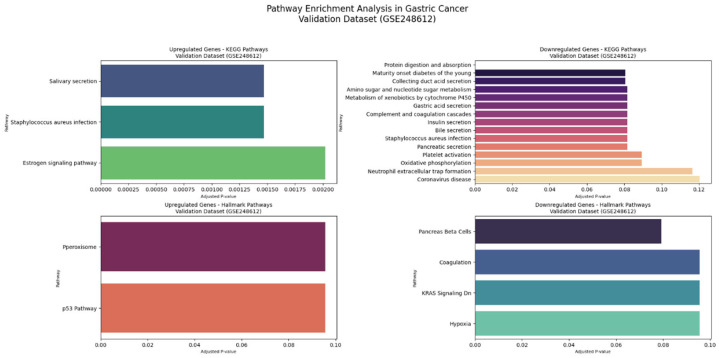
Comprehensive Hallmark pathway enrichment analysis in validation dataset (GSE248612).

The comprehensive KEGG pathway and Hallmark gene set enrichment analyses of intricate molecular alterations in GC across both datasets. Figure 9 shows the primary dataset (GSE26899), Comprehensive KEGG and Hallmark Pathway Enrichment Analysis. Figure 10 shows the validation dataset (GSE248612), Comprehensive KEGG and Hallmark Pathway Enrichment Analysis.

The primary dataset (GSE26899) demonstrated extensive pathway dysregulation. Figure 9 reveals upregulated genes in KEGG pathways, showing remarkable enrichment in ECM–receptor interaction (6 genes, *p*-value 1.01 × 10^−9^, odds ratio 76.61), focal adhesion (7 genes, *p*-value 3.85 × 10^−9^, odds ratio 39.65), and *PI3K-Akt* signaling pathways. The analysis of downregulated genes in KEGG pathways uncovered significant disruptions in fundamental cellular processes. Genes such as *COL1A1*, *LAMB3*, *ITGA3*, *FN1*, *SPP1*, and *THBS2* were central to these pathways, indicating significant extracellular matrix remodeling and cellular signaling alterations.

The comprehensive Hallmark pathway enrichment Analysis provides a detailed view of molecular programs in the primary dataset. The panel of upregulated genes Hallmark pathways revealed a profoundly significant epithelial–mesenchymal transition process, involving 15 genes with an extraordinary odds ratio of 160.46 (*p*-value 1.76 × 10^−24^). The panel of downregulated genes in Hallmark pathways further elucidated the complex molecular changes. Coagulation and angiogenesis pathways were significantly enriched, with genes such as *MMP7*, *SPARC*, *SERPINE1*, and *TIMP1* playing crucial roles.

In the validation dataset (GSE248612), KEGG pathway analysis for upregulated genes revealed three prominent pathways: salivary secretion (involving *CST2* and *CST1* genes, *p*-value 0.0009, odds ratio 54.67), Staphylococcus aureus infection (*KRT17* and *KRT16*, *p*-value 0.0010, odds ratio 53.49), and estrogen signaling pathway (*KRT17* and *KRT16*, *p*-value 0.0020, odds ratio 36.77). Downregulated KEGG pathways in the validation dataset demonstrated significant disruptions in fundamental cellular processes. The protein digestion and absorption pathway was most substantially affected, with *PGA3*, *PGA5*, and *PGA4* genes showing remarkable changes (with 3 out of 103 genes significantly involved, *p*-value 0.00002, odds ratio 85.24). Additional pathways included maturity onset diabetes (*NEUROD1* gene), collecting duct acid secretion (*ATP4A*), and various metabolic processes. Genes such as *NEUROD1*, *ATP4A*, *CHIA*, and *SULT2A1* played critical roles in these dysregulated pathways, indicating comprehensive metabolic reprogramming in GC.

The Hallmark gene set analysis provided deeper insights into molecular programs. In the validation dataset, upregulated Hallmark pathways were primarily associated with peroxisome-related processes, with *CRABP2* as a key gene (*p*-value 0.0508, odds ratio 21.45). The p53 pathway, involving *KRT17*, also showed significant alterations. Downregulated Hallmark pathways revealed disruptions in pancreas beta cell functions (*NEUROD1*, *p*-value 0.0198, odds ratio 56.84), coagulation processes (FGG), and *KRAS* signaling.

#### 3.4.3. Integrated Multi-Platform Pathway Analysis

The comprehensive multi-platform analysis revealed remarkable consistency across different analytical approaches, demonstrating the methodological robustness of our GC molecular characterization. GO, KEGG pathway, and Hallmark gene set analyses converged on several critical molecular mechanisms, providing a multi-dimensional understanding of cancer progression.

The analyses consistently highlighted key biological transformations. Nuclear division and cell cycle regulation emerged as primary points of convergence, with GO analysis emphasizing nuclear division and organelle fission processes, while KEGG pathway analysis revealed significant alterations in *PI3K-Akt* signaling pathways. These complementary findings strongly supported the observation of fundamental changes in cellular proliferation mechanisms.

Epithelial development and transformation represented another critical area of consistent molecular insight. The GO analysis prominently featured epithelial and epidermis development, which directly aligned with KEGG and Hallmark analyses’ emphasis on EMT pathways. This convergence provided robust evidence of profound structural and functional alterations in epithelial tissues during GC progression.

Extracellular matrix remodeling emerged as a third key area of concordant findings. Network analyses and GO terms highlighting extracellular processes were strongly supported by KEGG pathway discoveries of ECM–receptor interactions and focal adhesion mechanisms. Critical genes like *KRT16* and *KRT17* played central roles across these different analytical platforms, underscoring their significance in cancer molecular dynamics.

The consistent patterns across Gene Ontology, KEGG, and Hallmark analyses not only validate our initial findings but also offer a comprehensive, multi-dimensional view of GC’s molecular landscape. By demonstrating reproducible insights through different analytical approaches, this study provides a robust framework for understanding the complex biological mechanisms underlying GC progression.

These integrated insights extend beyond mere molecular description, pointing toward potential strategies for early detection, diagnostic targeting, and potentially personalized therapeutic interventions. The methodological rigor of cross-platform validation significantly enhances the reliability and translational potential of our findings.

## 4. Discussion

In this study, a comprehensive transcriptomic and machine learning-based approach was employed to identify reliable biomarkers and underlying molecular mechanisms in GC. By analyzing two independent datasets (GSE26899 and GSE248612) representing different expression platforms (microarray and RNA-seq), this work demonstrated the reproducibility and diagnostic potential of differentially expressed genes (DEGs) and their associated biological pathways. The integrative design and cross-platform validation ensure robustness and highlight the translational applicability of the findings.

The primary dataset (GSE26899) revealed 627 DEGs, with a predominant downregulation pattern in tumor tissues. This imbalance reflects the disruption of gastric functions in malignancy. Among the most significantly downregulated genes were *LIPF*, *PGA3*, *PGA4*, and *ATP4A*, which are involved in gastric acid secretion and protein digestion—core physiological activities of healthy gastric mucosa. The silencing of *ATP4A*, which encodes the gastric H^+^/K^+^ ATPase alpha subunit, is well documented in gastric carcinogenesis and is associated with intestinal-type cancer progression and glandular atrophy [25]. A recent experimental study further demonstrated that the reactivation of *ATP4A* through intragenic DNA demethylation effectively suppressed gastric tumor growth, indicating a direct epigenetic mechanism contributing to its silencing and therapeutic reactivation [25]. Similarly, PGA genes, encoding pepsinogens, are often repressed in gastric tumor tissues and considered molecular markers of gastric differentiation loss [26].

In contrast, a distinct set of upregulated genes was identified, including *CST1*, *KIAA1199* (also known as *CEMIP*), *MSLN*, and *TIMP1*. These genes are functionally implicated in protease inhibition, ECM remodeling, and epithelial invasiveness—Hallmark processes of tumor progression. *CST1*, a cysteine protease inhibitor, is overexpressed in GC and has been shown to promote tumor cell proliferation, migration, invasion, and activate Wnt/β-catenin signaling; it is also implicated in colorectal cancer proliferation via modulation of the Wnt pathway and as a let-7 target [27]. *KIAA1199* has been reported to be overexpressed in stomach cancer along with many other types of cancer [28]. *TIMP1* is significantly upregulated in GC tissues and promotes cell proliferation, migration, and invasion [29]. Notably, these genes also emerged as top features in the random forest classifier, strengthening their relevance as diagnostic indicators.

The machine learning classification approach implemented in this study demonstrated exceptional diagnostic performance. Using the top 200 DEGs as features, the random forest model achieved an AUC of 0.952, with a sensitivity of 96.4% and an accuracy of 93.5%. These results are consistent with previous applications of ensemble learning in GC, where random forests have shown superiority over linear classifiers in high-dimensional transcriptomic data [30,31]. Feature importance analysis identified *ADH7*, *TIMP1*, *KIAA1199*, and *ATP4A* as key genes contributing to discrimination. *ADH7*, involved in retinol metabolism, has been reported to have tumor-suppressive functions, and its dysregulation is implicated in gastric epithelial transformation [32].

Cross-platform validation using the RNA-seq dataset (GSE248612) further supported the reliability of the primary findings. Among the most strongly concordant genes across platforms were *CST1*, *KRT17*, *TIMP1*, *CHIA*, *PGA3*, and *ATP4A*. The consistent downregulation of *CHIA* (acidic chitinase), a gene linked to mucosal immunity and gastric inflammation, is particularly notable given its association with Helicobacter pylori-related gastric atrophy and cancer [33]. Additionally, the strong upregulation of *KRT16* and *KRT17*, keratin genes linked to epithelial stress responses and squamous differentiation, suggests structural remodeling and altered differentiation patterns in GC tissue [34]. These genes not only displayed significant log2 fold changes but were also functionally integrated in multiple enriched pathways.

Functional enrichment analysis of GO terms revealed significant alterations in nuclear division, epithelial development, and ECM organization—biological processes closely linked to tumor proliferation and metastasis. KEGG pathway analysis further underscored the dysregulation of critical oncogenic signaling cascades, such as *PI3K-Akt*, ECM–receptor interaction, and focal adhesion, all of which play pivotal roles in gastric tumor cell survival, angiogenesis, and invasion [34]. Among downregulated pathways, protein digestion and absorption, gastric acid secretion, and metabolic reprogramming were prominent, echoing the physiological loss observed at the gene expression level.

Hallmark gene set enrichment analysis offered additional layers of interpretation, identifying EMT as one of the most significantly upregulated pathways in the primary dataset. EMT is a key biological program that facilitates the transition of epithelial cells to a mesenchymal phenotype, promoting invasion, metastasis, and resistance to therapy. The involvement of genes such as *KRT17*, *MSLN*, and *KIAA1199* in EMT is consistent with earlier studies showing EMT as a driver of poor prognosis in GC [35]. Moreover, activation of the p53 pathway and suppression of *KRAS* signaling and pancreatic beta-cell functions further highlighted the complexity of molecular rewiring in tumor development.

Network-based integration of GO and gene–process relationships demonstrated that genes such as *KRT16*, *KRT17*, *CST1*, and *CRABP2* serve as highly connected hubs across multiple biological modules. These genes were not only significantly dysregulated but also contributed centrally to enriched biological processes, suggesting that they play integrative roles in orchestrating GC progression. Notably, *CRABP2*, a carrier of retinoic acid, has been implicated in cell differentiation and was upregulated in both datasets, supporting its potential involvement in epithelial tumor remodeling [36].

Taken together, these findings underscore the reliability of transcriptome-based biomarker discovery when complemented by rigorous machine learning and pathway enrichment strategies. The reproducibility of the results across different datasets and platforms confirms the methodological robustness and translational potential of identified genes such as *CST1*, *TIMP1*, *KRT16*, and *ATP4A*. These genes could serve not only as diagnostic indicators but also as potential therapeutic targets in personalized GC management.

## 5. Conclusions

This study demonstrates that integrating transcriptomic data from independent expression platforms, combined with advanced machine learning techniques, can reliably identify consistent biomarkers in gastric cancer. The findings highlight genes such as *CST1*, *TIMP1*, *KRT16*, and *ATP4A* not only as robust diagnostic markers but also as key players in the underlying molecular mechanisms, offering promising targets for personalized therapeutic strategies. By bridging molecular biology and clinical application, this approach provides new insights for early diagnosis and improved management of gastric cancer. Moreover, the cross-platform reproducibility and comprehensive functional analyses reinforce the translational potential of these findings, underscoring the value of combining bioinformatics and machine learning in uncovering novel targets for gastric cancer management and advancing precision oncology.

However, several critical points should be acknowledged. The use of AN tissues may introduce field effect bias, since these tissues can harbor molecular alterations due to their proximity to tumor sites. This may lead to an underestimation of expression differences when compared to normal tissues derived from healthy individuals. Additionally, due to the lack of clinical follow-up data in the public datasets used, survival analysis could not be conducted, limiting the assessment of prognostic biomarkers. Future studies should therefore aim to incorporate normal tissues from healthy controls and longitudinal clinical data to strengthen biomarker validation and to enable more robust prognostic assessments.

## Figures and Tables

**Table 1:** Top differentially expressed genes in primary cohort (GSE26899).

Gene Symbol	Log2FC	Adjusted *p*-Value	*p*-Value	Function	Regulation
*CST1*	3.04	1.82 × 10^−3^	2.15 × 10^−4^	Protease inhibitor	Up
*KIAA1199*	2.91	1.74 × 10^−6^	8.32 × 10^−8^	Hyaluronan binding	Up
*MSLN*	2.54	6.45 × 10^−3^	4.21 × 10^−4^	Cell adhesion	Up
*TIMP1*	2.41	3.12 × 10^−5^	1.58 × 10^−6^	Matrix regulation	Up
*KRT17*	2.35	8.94 × 10^−4^	1.23 × 10^−5^	Epithelial structure	Up
*LIPF*	−5.93	2.36 × 10^−7^	1.12 × 10^−8^	Lipid digestion	Down
*PGA4*	−5.86	1.74 × 10^−8^	8.25 × 10^−10^	Protein digestion	Down
*PGA3*	−5.76	5.91 × 10^−8^	2.80 × 10^−9^	Protein digestion	Down
*CHIA*	−6.15	3.24 × 10^−6^	7.41 × 10^−8^	Immune response	Down
*ATP4A*	−4.12	9.87 × 10^−5^	2.34 × 10^−6^	Gastric acid secretion	Down

**Table 2:** Machine learning classification performance summary.

Model	AUC	Accuracy	Sensitivity	Specificity	PPV	NPV	Cross-Validation
Random Forest	0.952	93.5%	96.4%	66.7%	94.1%	80.0%	5-fold CV
Neural Network	0.923	89.8%	92.7%	58.3%	91.4%	63.6%	5-fold CV
Logistic Regression	0.891	87.2%	89.1%	58.3%	90.2%	58.3%	5-fold CV

**Table 3:** Top upregulated genes in validation cohort (GSE248612) confirming primary dataset findings.

Gene ID	Symbol	Description	log2FoldChange	*p*-Value	Adjusted *p*-Value
3868	*KRT16*	keratin 16	9.66	2.00 × 10^−10^	3.11 × 10^−7^
3854	*KRT6B*	keratin 6B	7.71	2.30 × 10^−9^	2.80 × 10^−6^
105376938	*LOC105376938*	uncharacterized LOC105376938	7.53	3.38 × 10^−7^	1.18 × 10^−4^
1469	*CST1*	cystatin SN	7.32	3.40 × 10^−9^	3.63 × 10^−6^
100874364	*HOXC-AS2*	HOXC cluster antisense RNA 2	6.51	2.20 × 10^−9^	2.80 × 10^−6^
348825	*TPRXL*	tetrapeptide repeat homeobox like	6.41	1.42 × 10^−8^	9.89 × 10^−6^
3872	*KRT17*	keratin 17	6.13	2.00 × 10^−10^	3.20 × 10^−7^
1470	*CST2*	cystatin SA	6.09	1.00 × 10^−8^	8.58 × 10^−6^
101929694	*LINC02042*	long intergenic non-protein coding RNA 2042	6.08	1.36 × 10^−8^	9.89 × 10^−6^
1382	*CRABP2*	cellular retinoic acid binding protein 2	5.84	7.90 × 10^−9^	7.32 × 10^−6^

**Table 4:** Top downregulated genes in validation cohort (GSE248612) supporting cross-platform validation.

Gene ID	Symbol	Description	log2FoldChange	*p*-Value	Adjusted *p*-Value
27159	*CHIA*	chitinase acidic	−7.55	7.04 × 10^−5^	5.42 × 10^−3^
886	*CCKAR*	cholecystokinin A receptor	−6.62	1.00 × 10^−15^	2.90 × 10^−11^
5222	*PGA5*	pepsinogen A5	−6.47	1.62 × 10^−4^	9.76 × 10^−3^
643847	*PGA4*	pepsinogen A4	−6.14	1.24 × 10^−4^	7.97 × 10^−3^
6822	*SULT2A1*	sulfotransferase family 2A member 1	−6.08	1.00 × 10^−15^	2.25 × 10^−9^
643834	*PGA3*	pepsinogen A3	−5.98	1.61 × 10^−4^	9.75 × 10^−3^
124872	*B4GALNT2*	beta-1,4-N-acetyl-galactosaminyltransferase 2	−5.86	1.00 × 10^−15^	1.16 × 10^−9^
495	*ATP4A*	ATPase H+/K+ transporting subunit alpha	−5.28	9.27 × 10^−4^	3.31 × 10^−2^
4760	*NEUROD1*	neuronal differentiation 1	−5.15	1.07 × 10^−7^	4.96 × 10^−5^
2266	*FGG*	fibrinogen gamma chain	−5.13	4.61 × 10^−3^	9.84 × 10^−2^

**Table 5:** Cross-platform validated biomarkers with consistent expression patterns.

Gene Symbol	GSE26899 Log2FC	GSE26899 Adjusted *p*-Value	GSE248612 Direction	Validation Status	Primary Function
*CST1*	3.04	1.82 × 10^−3^	Upregulated (7.32)	Validated	Protease inhibitor
*KRT17*	2.35	8.94 × 10^−4^	Upregulated (6.13)	Validated	Epithelial structure
*TIMP1*	2.41	3.12 × 10^−5^	Upregulated	Validated	Matrix regulation
*PGA3*	−5.76	5.91 × 10^−8^	Downregulated (−5.98)	Validated	Protein digestion
*PGA4*	−5.86	1.74 × 10^−8^	Downregulated (−6.14)	Validated	Protein digestion
*ATP4A*	−4.12	9.87 × 10^−5^	Downregulated (−5.28)	Validated	Gastric acid secretion
*CHIA*	−6.15	3.24 × 10^−6^	Downregulated (−7.55)	Validated	Immune response
*KRT16*	2.89	2.15 × 10^−4^	Upregulated (9.66)	Validated	Epithelial differentiation
*CRABP2*	2.12	4.78 × 10^−3^	Upregulated (5.84)	Validated	Retinoic acid binding
*CCKAR*	−4.87	1.23 × 10^−4^	Downregulated (−6.62)	Validated	Hormone signaling

## Data Availability

The data presented in this study are openly available in the National Center for Biotechnology Information at https://www.ncbi.nlm.nih.gov/geo/query/acc.cgi?acc=GSE248612 (accessed on 7 May 2025), reference number GSE248612.

## Abbreviations

The following abbreviations are used in this manuscript:

GC	Gastric Cancer
GAC	Gastric Adenocarcinoma
GEO	Gene Expression Omnibus
RNA-seq	RNA sequencing
DEGs	Differentially Expressed Genes
GO	Gene Ontology
lncRNA	Long Non-Coding RNA
SNP	Single Nucleotide Polymorphism
EMT	Epithelial–Mesenchymal Transition
ECM	Extracellular Matrix
GSEA	Gene Set Enrichment Analysis
NCBI	National Center for Biotechnology Information
Padj	Adjusted *p*-value
log2FC	Log base 2 Fold Change
